# The Spas of England, &c.

**Published:** 1842-01-01

**Authors:** 


### THE SPAS OF ENGLAND &c. By *A. B. Granville,* M.D. 3 vols. octavo. Colbourn, 1841.

DR. GRANVILLE is one of the first living locomotives that now exists. With great talent for observation, his tablets are ever ready to receive and record the transient impressions on a lively imagination. Nothing escapes his notice. From the tariff of a dull French "Poste," to the magnificent bill of fare at a splendid table-d'hôte in a Cursaal—from a cold and bitter draught of Pullna, drawn from a ditch in Bohemia, to a goblet of the boiling Sprudel, or the sparkling Kreuzbrun—from a musical diagram of the prattling echo on the Rhine, to a profound speculation on the central caloric of the globe we inhabit—from an architectural criticism on the façade of a terminus to a geological disquisition on a Druid's circle—all these, and every thing else which science discovers, art invents, or the press commemorates,

"Quicquid agunt homines,"

contribute to fill the portfolio of our lively and indefatigable author.

The "SPAS OF GERMANY" having gone off with such eclat, it was natural to expect a pendent or sequel to them, collected from the "SPAS OF ENGLAND." But here, we suspect, the worthy Doctor calculated without his host—or perhaps the HOST HIMSELF overshot the mark. The old Scotch proverb that "far fowls have fair feathers," told much in favor of the foreign waters, whilst the homely adage that "familiarity breeds contempt," told equally against the "Spas of England." But independent of these proverbs, our comparative ignorance of the German mineral waters rendered a description of them agreeable, a little *embellishment* pardonable, and a desire to drink them almost irresistible. The consequences might have been foreseen. The gossip and travelling anecdotes of a foreign tour will go down much easier than descriptions and scenes at our home watering-places, with which we are all familiar. The only judicious plan was, in our humble opinion, to throw overboard all the gossip and anecdote, and thus compress the real and necessary information respecting our own medicinal springs, into one volume, instead of spinning it out into THREE. This might have been done with the greatest ease, had Dr. Granville possessed the "organ of concentrativeness" in greater development—or the "organ of self-esteem" in less. The author, or rather the publisher, ought to have *commenced* with "the Spas of England," as he *ended* with those of Germany—by reduction of size and price to *one-third* the original dimensions and amount.

The three volumes before us are extremely well calculated for the operation of a condensing engine in the hands of a practised analyzer, since the materials are so *squeezable* that they might be reduced to almost any narrowness of limit, not only without injury, but with the greatest advantage. Such an article would gain in strength in exact proportion as it diminished in size, while its utility would be inversely to its verbosity. We do not entirely blame our author for taking a course directly the reverse. When a writer is paid by the sheet, and a publisher is remunerated by the volume—when, in short the author cannot *dictate*, without danger of remonstrance, to the man-midwife who delivers his brain of its literary offspring, the case will ever be, as it has always been, that *trade* domineers over *literature*, and gold over reputation. This, however, is somewhat out of our way—a mere "sat verbum." We must now to our work.

We must pass over the several pages of "Popular Considerations on English Mineral Waters," with an admiration of the extremely shrewd recommendation at page 47—namely, that all patients who are thinking of visiting a Spa, whether at home or abroad, should put this home question to their doctor—have you personally visited the proposed Spa? This is a *popular* hint worthy of being recorded in letters of gold!

### CHAP. I.—"CLEARING THE WAY WITH THE PUBLIC."

This consists of a dialogue (imaginary) between a SPA-DOCTOR and his friend, with which the public has no more concern, than with the "dialogues of the dead."

### CHAP. II.—"RAIL-ROAD TRAVELLING."

Dr. G. comes to the conclusion that "such a mode of conveyance is not more likely to do mischief to people's health than any other hitherto adopted." But he makes a qualifying declaration that—" Constituted as rail-roads are at present, * * * * it is not impossible that some easily affrighted dame, some highly nervous old gentleman, &c. may suffer from railway-travelling, or from some of its concomitants." Few will hesitate to subscribe to this doctrine—and all will hope that Dr. Granville's improvements will be adopted throughout England.

CHAP. III. and a rather long one, is on the same subject—a division which is of no interest or concern to any one but the compositor, who calls a blank page a "piece of fat." It is fat to him, but it is monstrously lean to the reader! We were a little surprised at the following statement:—" To a traveller who is in a hurry, and desires *to enjoy as many of the comforts of a rail-road as he can procure, the night-train* is unquestionably to be preferred." p. 27, vol, 1. All we can say is—de gustibus non disputandum.

Passing over the moving scenes in Euston Square and the Birmingham station, the first of which is pronounced to be " dramatic in effect," and the second " no less theatrical," we will transport our readers at once to

### HARROGATE.

CHAP. V.—Dr. G. very justly remarks, that " Harrogate has the very *air* of a watering-place." No one will doubt this, or ever forget the *air* of it, who takes a good large glassful of the Old Sulphur Well. If any one can imagine an old rusty gun-barrel (that had not been fired or scaled since the time of the Spanish Armada) to be well scoured out with sea-water, in which a large number of rotten eggs are dissolved, and, last of all, a stream of sulphuretted and carburetted hydrogen gas directed through the "preparation"—he will have a good idea of this " Yorkshire Stingo" at Low Harrogate.

But the more stinking a medicine is, the greater will be its reputation—and Harrogate is now becoming one of the most renowned spas in England. It is a place of many springs, and so many chops and changes have taken place in the names of the wells and of their proprietors, that it is no easy matter to ascertain them. It took us forty-eight hours, in the month of August last, with Dr. Granville's, Dr. Hunter's, Dr. Scudamore's, and other books in our hands, before we could unravel the intricacy that prevails here. Here we have, at Low Harrogate, two sulphur wells, (the old one without a pump, and the Montpellier well with one) three salines, (the Montpellier Cheltenham, Walker's Strong Saline, and the Promenade Cheltenham Saline,) with two extensive bathing establishments. Between Low and High Harrogate, in a swampy common, we have the oldest well of all—a pure chalybeate—The TEWIT, with a mere rude stone shed to defend it from the rains and winds, and attended by a very ancient Naiad, who doles out the clear steel spring to a few votaries. Near it stands the ruins of a cottage, which give the place a triste air of solitude and desolation!

" Deserted in its utmost need
By those its former bounty fed."

A narrow road, on which two gigs could scarcely pass, leads from Low Harrogate to the original spring which first gave this watering-place " a local habitation and a name ! ! "

Ascending about a quarter of a mile on the common, we come to the second spring, in age, called the " Old or Sweet Spa," situated nearly opposite the Granby Hotel, in High Harrogate. Some more attention has been paid to this pure chalybeate, as it is enclosed in a stone building, and open to all bibbers, who can bring a glass or cup with them. Both this and the Tewit are pure chalybeates, one containing two grains, the other two and a half grains of iron in the gallon—or about a quarter of a grain in the pint.

Traversing the table land or common of High Harrogate, one mile and a half from the Old or Sweet Spa, on the Knaresborough road, we come to the Starbeck Wells—one, a mild sulphur, and the other a mild saline chalybeate water, with a small suite of baths.

Thus then, we see that there are eight or nine drinking wells at Harrogate, all varying more or less from one another in their chemical composition and medicinal effects. Excepting the two pure chalybeates, all the waters are aperient. The sulphur wells being most so, though containing little of other aperient salts than the muriate of soda.

The " Montpellier Saline Chalybeate " contains 110 grains of salts in the pint, of which muriate of soda makes 81· grains—sulph. sodæ 2½—muriate of lime 22—muriate of magnesia 4 grains—oxyde of iron nearly half a grain.

The " Royal Promenade or Cheltenham Saline," as it is called, contains in the pint, as follows :—muriate of soda 24 grains—muriate of lime 18 grains—muriate of magnesia 10 grains—carb. soda 1½ grain—oxyde of iron half a grain.

The impropriety of giving the term " Cheltenham " to this water is evident—there being no sulphate of soda or magnesia in it. Its aperient qualities must therefore rest on the muriate of soda, and muriate of magnesia.

Walker's Pure, or " Strong Saline," as it is termed, contains about 90 grains of saline matters in the pint—into which enter 76 grains of muriate of soda—6 of carb. soda—5½ muriate of lime—2 of muriate of magnesia.

We need not specify the wells any farther. They are capable of application to a great variety of complaints, whether taken internally or used in baths, or both conjoined.

Among our forefathers the Harrogate waters worked wonders. Dr. Dean informs us that " the common people drink them, and they expel *reef and fellon*. They soon help and cure by washing and bathing, itch, scab, morphew, tetters, ringworm, and the like."

It appears by the testimony of Dr. Neale of Leeds, that the Old Chalybeate, or " Sweet Spa," as it was called, was not behind the sulphur wells in his day.

" As to the virtues of this spring there is scarce any disease incident to mankind wherein its inward or outward use may not be of service. I have been an eye witness of its effects nearly forty years, and I have not neglected drinking it myself any one season all that time; and though I am now in my 66th year, yet I am strong and vigorous, free from the complaints of old age. But because a general and just commendation of this spring will not be satisfactory, without condescending to enumerate the diseases wherein it's proper.—It's good therefore, to restore a lost appetite and digestion, to mitigate the scurvy, correct all acid humours in the lympha, blood, nervous and pancreatic juices. It cleanses the kidnies and ureters of slime, sand, gravel, and great stones, and is very assistant in curing ulcers in those parts. It removes the hyppo's melancholy, opens obstructions of the lungs, liver, spleen, mesentery, and glands. It purifies the blood, and renders the spirits in the body more cheerful and lively. Several short-winded, ashmatic, weak, and lame people, have had their lungs and limbs restored to their former strength and usefulness. It relieves inveterate headaches, especially if at the same time you use the cold bath. It is also very serviceable in the gout, by restoring the use of lame hands, knees, legs, and feet. It revives the memory, clears the brain from viscous humours, and helps the eyes by drying up rheums. It relieves sharpness of urine, strangury and disury, if there is no large stone or other stoppage in the urinary passages. It corrects acidity in any part of the body; as in the heartburn, belchings, sourness at the stomach, gripes, cholic, and borbarigmos. It opens the breast and lungs, cuts tough flegm, promotes expectoration, and has often been successful in the cure of blood-spitting, hectic fever, too great heat and dryness of the skin and body." 86.

But descending along the stream of time to our own days, we shall give an extract from one of the most recent writers—Dr. Adam Hunter, of Leeds, who has enjoyed means of observations which could not fall to the lot of any casual visitor of this celebrated spa.

" Although well aware that nosological arrangement has latterly been very much disregarded, and even discouraged by some celebrated lecturers, I have long been convinced of its utility, not only in the study but practice of medicine. Allowing that in many instances the classification is imperfect, and founded upon hypothetical reasoning, the abstract question of its correctness seldom interferes, or will soon cease to have any influence over the mind of the practitioner; while the arrangement of observations under their respective heads, greatly facilitates inquiry, and gives accuracy to the deductions obtained. With this view, as intended for the medical reader I shall briefly glance at the nosological arrangement of Cullen, in relation to these waters. As alterative aperients, they will be found chiefly applicable in the chronic forms of most of the diseases included in the class pyrexiæ and order phlegmasiæ; in the sequelæ of most of the exanthemata; and in the hemorrhagiæ. In class second, neuroses, order second, adynamiæ, these waters possess great power. In almost every one of the third class cachexiæ, they are no less useful. Likewise in a considerable number included in the fourth class, locales.

The *Sulphur Water* speedily and safely carries off the effects of intemperance in those who, having spent the winter and spring in festivity, resort to Harrogate with their system loaded with impurities, from the excesses of the table, and whose stomachs are debilitated by these and similar causes. Its use is acknowledged in those predisposed to apoplexy. In chlorosis or green sickness, it has been usual to drink the sulphur water for some time, and then take the chalybeate. In diseases of the skin, especially the order squamæ of Willan, who mentions his having seen some very obstinate cases of lepra, alphos, and psoriasis, completely cured by this water; in porrigo, herpes, and the impetigines; scrofula, scurvy, secondary syphilis, and ulcers, its use has been equally efficacious. In gout also, in both its principal divisions of regular and irregular, or atonic; in the first, the constitution is sound and vigorous, the fits are severe and regular, and there is generally plethora and inflammatory diathesis: in the second, the constitution is debilitated and diseased; the fits irregular; the alimentary canal, head, breast, and urinary passages, affected with various complaints, alternating with the fits. In the former the water may be taken as an habitual laxative; in the latter, its use requires considerable caution, the warm or vapour bath in conjunction with it will frequently prove useful. In the numerous list of complaints now comprehended under the term dyspepsia, or indigestion; in many of which, however, the saline chalybeate water is preferable. In flatulent and bilious cholic, habitual costiveness; hypochondriac affections; jaundice; hemorrhoids or piles; worms; in chronic reumatism, with the warm bath; and lastly in some cases of dropsy, by active purging. In stone and gravel, the weaker sulphur water at Starbeck has been much extolled.

Having already detailed the properties of the *Saline Chalybeate Water*, its general effects will be readily understood. In most of the diseases already mentioned, where the sulphur water is found to occasion relaxation and weakness, or where the strength is not recruited under its use, this will be found a proper substitute. In all female weaknesses, I am disposed to give it the preference. In chlorosis; atonic gout; hepatic and nervous affections; diabetes, and some cases of tic doloureux, it has likewise been of the greatest advantage.

The effects of the *Saline* or *Leamington Waters* have likewise been stated, it is unnecessary therefore that I should further advert to them.

The action of the *Pure Chalybeates* is more distinctly tonic than either of the former. In all cases, therefore, where the system is relaxed, these waters properly administered, produce the happiest effects. When the sulphur or saline waters have carried off the previous obstructions, a short course of one of the chalybeate springs will brace the solids, and give tone and vigour to the system." 133.

Having, for many years past, been in the habit of sending patients to Harrogate and other mineral waters, we have, to use the language of anatomists, a kind of *distal* experience of their medicinal effects; and we can very much corroborate what Dr. Hunter has stated respecting Harrogate. We are inclined to think, however, that this Spa owes a good deal of its celebrity to the air as well as the waters. We have seldom experienced more bracing or exhilarating effects in any locality, than at High Harrogate. The air of the common there is remarkably clear, pure, and invigorating, and some of the hotels, as the "GRANBY" (a first rate) and "GASCOIGNES" (a second rate but most comfortable one), are well situated for those invalids who are able to walk or drive down to the wells in Low Harrogate in the mornings.

There are several LIONS in the environs of Harrogate, to which excursions are made—the BRIMHAM rocks, ten miles from the spa, being that which most interested me. This is a huge collection of BOULDERS, of all shapes and sizes, scattered over the top of a hill, and along its sides, in inextricable confusion. In some of the Guide-books they are termed "Druidical"—a piece of sheer nonsense. These rocks are composed of sand or gritstone—were deposited at the bottom of a diluvian or antediluvian ocean—raised up by fire or some terrestrial convulsion—then subjected to mighty currents or torrents, which wore them away *horizontally* (not *vertically*, which winds and rains would have done), and last of all, they were laid bare, either by the retreat of the waters, or the elevation of the strata on which they reposed. There they now stand, or lie, or rock about on peduncular bases, having assumed the most fantastic shapes, and been christened by appropriate names accordingly. Some have thought that this "stony forest" was merely a collection of boulders fortuitously rolled together by diluvial tides or currents. But had this been the case their strata would not all have had the degree of horizontality, which they now have, except where a rock has evidently fallen out of its place, or rolled down a declivity.

Before quitting Harrogate I may just allude to a very curious kind of malady with which it has affected two or three spa-tourists of the medical tribe. The name is not in Cullen's, nor even in Good's Nosology—viz. "PLUMBO-PUMPO-PHOBIA," or the dread of lead in the pump-rooms of English Spas. Sir Charles Scudamore seems to have been the first who felt its effects at Harrogate; but the disease was of a mild character, and a few dips in the Buxton-baths cleared the worthy Doctor of his PLUMBO-PUMPO-PHOBIA. Not so with Dr. Granville. He had such a violent attack of this rare malady that it required several large draughts of Pullna water (without which the Doctor never travels) to produce the "crise," and expel the poison. We have understood that Dr. G. still feels the effects of the PUMPO-PHOBIA, and avers that such a disease exists not at any of the German Spas—for this good reason, that no pumps are there employed for raising the medicinal waters from their lowly beds.

Now we would recommend Sir C. Scudamore and Dr. Granville—and indeed all other plumbo-pumpo-phobic doctors, to examine some of the leaden pipes through which the mineral waters run from the numerous wells near the Lansdown Crescent, Cheltenham, to the Rotunda and the laboratory for making the salts there. If one of the pipes be broken, it will be found to be lined by an insoluble incrustation or amalgam that completely defends the water and the lead from any action or re-action on one another.* But admitting that this were not the case, we will venture to affirm that all the lead swallowed in a whole season at Harrogate or Cheltenham, would not exceed the dose which an individual might take at once, without any necessity for resorting to the stomach-pump. And what becomes of the millions in London and other towns, who imbibe water, porter, and other fluids through pipes and pumps, from year to year? The PLUMBO-PUMPO-PHOBIA is a chimera.†

And let us observe here that the nakedness and simplicity of an open well are not entirely free from danger, or the idea of danger. Any scoun-

---

* Dr. Christison observes that this crust on the inside of leaden tubes through which saline waters run, is at first carbonate of lead, and the tube gains weight; but after a time it is changed, and adheres with great tenacity to the lead beneath. "The most careful analysis, says he, cannot detect any lead in the water, or floating in it, or united with the insoluble matter left on the side by evaporation. In short, the preservation of the lead from corrosion, and of the water from impregnation with lead, is complete." P. 482.

† Dr. Christison, in his admirable work on poisons, has shewn that the presence of neutral salts in water completely deprives it of the power of holding lead in solution. So great is this protective influence, that an almost inconceivably small quantity of phosphate of soda, or hydriodate of potass in solution, will prevent the corrosion of lead! How, then, are the Cheltenham or Harrogate springs, so impregnated with neutral salts, to carry lead into the stomachs of the drinkers?

drel may throw a pound of arsenic, or other deadly poison into the old well at Harrogate, by night, and send twenty or forty bibbers to their graves next day, without fear of detection? If villains will throw logs of wood on a railway, to kill people by the score or the hundred, they may be capable of poisoning wells at a watering-place. These and other considerations have brought the Harrogate corporation to the resolution of raising a pump-room over the old sulphur well, and drawing up the water by a pump, as is done in the Montpellier wells. What will Messrs. Scudamore and Granville say to this?

It will be seen that we have extracted little or nothing from our author, having examined the spa with our own eyes, and drank copiously of its fountains. We shall here, however, quote a favourable sample or two of Dr. Granville's manner and matter—and if the Doctor had indulged less in gossip, and more in practical remarks, like the following, his volumes would have escaped censure, and secured to themselves a very extensive sale.

"At present, I applied myself with a serene countenance and an empty stomach to the quaffing of a large tumbler of the fetid stream, previously warmed by mixing with it some of the same which is kept constantly heated in stone jars placed on the top of a fireplace.

The water is perfectly colourless and transparent, and almost brisk from the escape of gas. The first impression on the tongue is intensely salt, followed by the peculiar bitter taste of salt water, but leaving an *après-goût* like that which remains after chewing bitter almonds. It goes down oily, and at the temperature at which I drank it (115°) the sulphureted gas is scarcely perceptible. I repeated the same quantity four times, diminishing each time the artificial temperature until I drank it cold, thermometer then marking 52°, while the external atmosphere was at 60°, and the nauseous taste had increased with the descending temperature.

The whole quantity I took in four times, I noticed people to drink at twice only, and quite cold. Writers on this water have recommended the latter practice. This is an error which I was sorry to see committed at all the English spas. There are few stomachs which can bear with impunity the weight of two doses of three-quarters of a pint each of a cold, salt, and sulphureted water, drunk with a short interval between. Few stomachs can stand the slow extrication of the imprisoned gas, which, once ingested with the cold water, is gradually disengaged by the warmth of that organ. It then mounts into the head, and produces a confused, heavy, and unpleasant feeling.

This I have put to the test of my own personal experience. Drunk quite cold, I found the water particularly heavy on the stomach, and in an hour's time my head ached not a little. Some of my younger patients in this place experienced similar effects; and, indeed, upon inquiries among strangers, who were religiously following the recommended practice, I ascertained the case to be precisely the same. At all events the first glass or two should be warmed, but not so much so as to drive off the whole of the sulphur gas.

With respect to quantity, that point has been determined by long experience, and by very competent authorities. It did not appear to me that people on whom devolved the management of the water, at the several spas I visited in this county, were sufficiently aware of the importance of this consideration. The quantity drunk, at one time, should be such that during the fifteen minutes' walk, which is to elapse between one dose and the next, the stomach may nearly have got rid of the first before it receives the second. Four ounces of liquid ingested will nearly disappear from the stomach in the course of twenty minutes, particularly when assisted by walking exercise."*

This was at the Montpellier sulphur well—the following passage relates to the OLD WELL.

"To that well I next extended my trial of the water upon another morning. Sallying out for that purpose at 8 A. M., I found that 'holy temple of Hygeia' thickly surrounded by people of the middle class. No contrast can be stronger than that which exists between the sort of people seen to quaff large goblets of the water here, and at the subscription-rooms; and the old women, nearly in tatters, placed in a row behind a species of stone parapet, over the top of which, as upon a counter, the salutary fluid from the well is delivered to every applicant, formed a curious sight to one who had been accustomed to behold the neat, pretty, and alert handmaidens of the Sprudel.

These noisy creatures (who look like so many fishwomen) administer the water either cold, or mixed with some previously heated, and in tumblers, in size nearly a pint. The well is behind them, and there is no lack of the water, which is squandered profusely at no charge whatever, save perhaps a solitary copper-piece, which some one, more generously disposed than the rest, throws with an air of protection on the stone counter.

I quaffed twice of this second sort of sulphur-water, about six ounces each time, and warm. It goes down as oily as the Montpellier, is not quite so intensely salt to the taste, and is decidedly without that *après-goût* of bitter almond which I think is a pleasant feature of the sulphur water at the Montpellier; and which, in the latter case, remains adhering to the tongue for some hours after drinking it.

Like the Montpellier, the water of the old-well, especially if taken cold, troubles the head, and some days gives the headach; it produces eructation of sulphureted gas, as when one has been eating half a dozen eggs boiled hard, or in a *worse* state; it acts promptly on the kidneys, and seems to promote the action of the intestines, as well as to influence the character of its secretions in the course of the morning. When neither of these effects are produced, the water is not properly digested, and the head is the more affected." 54.

Whether the hygeian nymphs had been changed by Dr. G.'s animadversions, we know not; but certainly they were very far from being either "noisy" or looking like "fishwomen" when we were there. They were decent in their dress, civil in their manners, and quiet in their demeanor. On several successive mornings we visited the old well, as well as the others, in the middle of August, 1841, and never found less than ten or a dozen of women baling out the water as fast as they could to their expectant visitors. At no hygeian fountain on the Continent did we ever see half that number necessary for supplying water to the drinkers. This is saying a great deal for the reputation of Harrogate.

Table-d'hôtes are general at the hotels here, and living is moderate. Two physicians are resident on the spot—Dr. Kennion, the successor of the worthy Mr. Richardson—and Dr. Bennett. As the hospital stands on the very brink of the peat-bog, through which rise several sulphur springs, mud-baths might be easily prepared, and employed at the hospital in obstinate rheumatic complaints, and old-standing and inveterate

* Vol. I., p. 52.

cutaneous affections. But we were told that there was no chance of their coming into use at Harrogate. The more the pity.

Upon the whole, we left Harrogate with most favourable impressions as to that spa. Its capabilities are so great, and its springs so various and numerous, that it cannot fail to stand at the very head of British mineral waters in future.

We are unable, at least for the present, to follow our lively and velocipede author to the numerous—we had almost said, numberless, minor spas which he visited—almost discovered (that is, " brought to light ") north of Harrogate. Should the ensuing " dog-days " release us for a month or two from the " dog-cart," we may march north to see the lions which Dr. Granville has shewn up to the public and the profession. In this article we shall only accompany him to some of the principal spas, with which we are personally acquainted.

### 2. Matlock.

The site of " Matlock-baths " is one of the most romantic in England, or in the world. The banks of the Derwent closely resemble those of the Elbe in Saxon Switzerland—except that they are more beautiful, and less wild and barren. If mineral waters be " animated beings," as many of our German confreres believe, they are very fortunate ones. If they contain active and powerful ingredients, like Carlsbad, Marienbad, Harrogate, &c. &c. their reputation in the cure of diseases is elevated on that account. If, on the other hand, they are nearly free from medicinal substances, then they are renowned for their purity, and consequently their remedial efficacy. Where they are neither strong nor pure, but intermediate, there is their mysterious vitality, caloricity, telluricity, or some hidden property communicated to them in the bowels of the earth, which gives them the power of working miracles when they see the light.

If the waters of Matlock, which are pure waters with the dead chill slightly taken off, possessed medicinal virtues, they must have been beholden for them to occult agency or homœopathic operations. They are used as common drink by the inhabitants, and exhibited gratis to the guests of the hotels. They taste soft, light, and pleasant. We took a bath raised to 96°, and found it very agreeable to the skin. A very few grains of saline matters—magnesia, lime, and soda, neutralized, are all the medicinal agents the Matlock waters can boast of.

Matlock is often selected as an agreeable sejour in the Summer or Autumn, on account of the pure air, wholesome water, romantic scenery, and interesting excursions. We descended into some of the caves and caverns—and ascended to the highest elevations. The contrast is great —and much in favour of the latter. The exploration of the dark and damp spar-caves is by no means a

" facilis descensus Averni "

and the " revocare gradum " is still worse.

### 3. BUXTON.

The drive, of some twenty miles, from Matlock to Buxton, through "Anglo-Saxon Switzerland," presents some of the best and most picturesque scenery which I have seen in England. The banks of the Avon, about Clifton, however, afford a formidable rival.

Buxton itself is now a lion of considerable magnitude among the spas of England, though formerly it presented little else than barren heaths and a gelid climate, now converted into undulating hills and dales, with wood and water, corn and meadow, river and rill—the whole forming quite a riant prospect. The air of this elevated region is bracing, and, like that of High Harrogate, imparts elasticity to the body and hilarity to the mind of man, There are a sufficiency and variety of agreeable walks for the accommodation of invalids. Buxton is more than a thousand feet above the level of the sea, and although much rain must fall in this elevated region, yet the air is not damp—colds are seldom caught—and epidemic diseases are unknown.

The Romans never failed to hunt out thermal springs wherever they existed. Although the temperature of these waters (80°) was not quite what the greasy soldiers and luxurious officers wished, yet they erected baths here, and various ruins and vestiges have been discovered confirmatory of the fact. The modern bather in these tepid floods must derive great satisfaction from the knowledge that he is plunging in the same spring where the weary limbs of Mary Queen of Scots had been laved, as well as those of Leicester, Burleigh, and other stars of their day. Useless crutches were here hung up, and numerous votive tablets erected in gratitude for the *miraculous* cures performed by the holy waters of St. Anne, the tutelar saint. But when the reign of fanaticism came, in the days of the Commonwealth, these emblems of *Popery* were demolished, and the springs themselves anathematized and outlawed! When the storms of persecution blew over, the Buxton waters gradually regained their celebrity, and the Dukes of Devonshire proved themselves to be the best tutelar saints, by erecting splendid buildings round the spas—improving the roads—planting the hills—and cultivating the vales.

The baths are public and private. The public one in which we saw several gentleman bathing and swimming, quite naked, would accommodate ten, fifteen, or twenty people, (male or female,) very well in this way. A large and powerful pump, or douche, is kept in constant employment. The following description of the public bath, though a little too higly coloured in some places, is, upon the whole, correct.

"In the public bath I saw many people bathing, three or four at a time, and several in succession. The operation with most of them was expeditious, the greater number of the bathers remaining but two or three minutes in the water, and being always in motion.

The water, from the dark colour of the rock at the bottom, and the darkness of the dome, (for it is in a vault under 'the Old Hall,') looks, at first view, dingy, and greenish; but it is as limpid, transparent, and colourless, as the one I drank at the spring. The form of the bath is an oblong square. The water surges about the middle, near the outer wall, to the height of four feet, and passes off at one of the extremities of the bath by waste pipes. Bubbles of air may be seen rising in succession from time to time. At other times a single one, much larger than the rest, will come up, to break at the surface.

The people bathing differed, it appeared to me, in opinion as to the impression made on them by the water. Some said it was very cold; others declared it was very comfortable. As the sun darted a slanting ray through the half circular window close to the vaulted ceiling over it, the surface of the water exhibited a gathering of scum, having an unusual appearance in any mineral water, which took away from me the temptation I had at first experienced of trying the effect of the Buxton water at this nearest point of its source.

The overflowing or escape of the surplus water through the waste pipes, is never so quick but that the said scum, or floating matter, remains too long spread over its surface,—as I witnessed during the half-hour I kept watching the proceedings of those who were in the bath. Indeed, one of the attendants comes now and then with a broom, and sweeps from off the surface the coarser particles, and thus restores to the water its natural appearance. But, at best, this is but mixing up with the water, or dissolving in it, the objectionable substance.

Altogether, the bathing in such a *piscina* was not such as to please my fancy; and when I beheld the class of persons, too, who kept coming in (for the access is free, and the bath always open), and their dress and appearance—when I saw the pot-bellied farmer of sixty, half palsied, and the lame artisan with his black and callous hands, and the many who suffered from cutaneous disorders—all plunging together, or one after the other, in quick succession—some of whom would set about scrubbing from their hardened cuticles the congregated perspiration of ages, with a hand-brush kept *pro bono publico* on the margin of the bath;—I say, when I beheld all these things, I confess my courage failed me, despite of my constant desire to try on myself, and ascertain by my own feelings, the effects of the various mineral waters I have examined."—*Granville,* p. 65.

The gentlemen's and the ladies' private baths (natural temperature) are at some distance from the source, and are cooler. We did not bathe in these but in the public bath, and certainly perceived very little shock on the first immersion. In a minute or two, the water felt very comfortable. The following is Dr. Granville's description of the effects of the private bath at natural temperature—or rather two or three degrees below 82°.

" I entered the bath about twenty minutes before eight, A.M., my pulse at eighty-two. I had drunk half a pint of the mineral water some time before. The immersion was by the steps, and therefore gradual. The feeling of cold on the skin produced by the first approach of the water formed a striking contrast with the pleasing warmth of the atmosphere of the room. When I let myself down into the middle, and at the bottom of the basin, by holding the chain which hangs from the centre of the ceiling, the shock was precisely similar to what I have often felt when plunging into the open sea at the same time of the day and year. It took my breath away, and tightened the thorax, producing, however, not the slightest vestige of disturbance, either in the head or in the movements of the heart.

I partially got out and recovered my breath, and again plunged into the bath, all within two or three minutes. The water felt still cold, but not so as to affect the respiration this time. After the first four minutes, I being either standing upright on the tiles which felt cold to the feet, or floating horizontally under the water, a degree of warmth began to pervade the body along its surface, and was evidently on the increase at every half-minute. The skin felt soft—not puckered nor corrugated in any part, as is generally the case in warm water, and many mineral springs; when the hands were passed over the body, they glided readily over it.

Even after a few minutes longer, I experienced no inclination to sleepiness, and the head continued in the same state as when I went in. Before I left the bath, however, I ascertained that, contrary to the effect produced by an ordinary warm-bath, if I raised my limbs from the middle of the depth to nearer the surface, the difference of feeling, as to warmth, was what I should have estimated at about three or four degrees of increased temperature.

So much so was this the case, that quitting the position in which the limb was previously stationary, and around which the water felt as if it had grown cold, and raising it to the position before alluded to, I could have imagined that I had placed my limb into a totally differently-heated water—into one, in fact, of a regular tepid bath, so genial was the first impression. But then it was only a *first* impression, which soon vanished, to be again renewed by seeking with the limbs, or any other part of the body, another and a new position.

At the expiration of about ten minutes, I might have fancied myself, from my own feelings, in a bath of 94° or 95°, or in a regular tepid bath; and this apparent feeling or impression was even stronger if I got on the steps of the reservoir, and quite out of the water, and immediately plunged into the water again.

Judging from this single experiment, which I have detailed minutely for very obvious reasons, I should say that the proper mode of using these baths would be, not to plunge, but to walk gradually and quickly into the water up to the chin, and out of it as quickly again. This operation should be repeated at least three or four times, occupying perhaps two minutes each time in doing it; and lastly the bathers should return into the water for the sake of a real bath, which would then produce pleasurable sensations, and could be borne very well and quietly for ten minutes longer, or even a quarter of an hour,—during which time, the body would receive the full benefit of these volcanic waters.

There is no disguising the fact: Buxton is a cold and not a tepid bath, and only becomes tepid to the feelings by a little time and management—the same as in the open sea—but not in an ordinary water-bath at 83°. The difference here is, that the warmth, when once felt, is a permanent sensation, were you to remain even hours together in the bath; whereas in ordinary water, tepid bathing, or the open sea, or in a river or a spring in the sun, the water which at first might seem tepid, would soon progressively feel colder and colder." 39.

When we consider that these waters contain only about two grains of saline matters in the pint, we can hardly suppose that they lose much of their medicinal agency by being heated by steam to the temperature of the body, or even of the blood. Dr. Granville, however, is of a very different opinion. "The most marked effect of the Buxton water (says he) is that of stimulation, whether the water be taken internally or used externally." And a little farther on he affirms that—" the fact is, again, that the stimulation is due, not to *thermometrical* heat, but to the portion only of *telluric* heat inherent in the water." This statement we cannot contravene, not having been *deep* enough in the bowels of the earth to distinguish between thermometrical and telluric heat. All we can say is, that in bathing at a temperature of 98° in the private baths, we experienced as agreeable sensations as we ever did in the Serpent's Bath, Wildbad, Tepliz, or Pfeffers. The lubricating quality of the Buxton water is very peculiar. When we rubbed the hand over the surface of the body, or against the tiles, the parts felt oily—or as Dr. Belcombe of York once characterized it to us—it was like bathing in new milk. The water itself is beautifully clear. We quite agree with Dr. Granville in the following remark. " It was beautifully transparent, and of a faint aquamarine colour. The minutest object could be perceived, but magnified considerably. The very lightest coloured hairs on the arms appeared dark from increased size, which seemed double their natural one at least." When the bath was cleared out, Dr. G. observed a kind of "oily slime" adherent to the sides; and yet he avers that the skin of the body felt *rough,* when the hand was passed over it in the bath. This was the very reverse of the feeling which we experienced—a feeling corroborated by every one with whom we conversed on the subject. Let those who plunge into the warm waters at Buxton decide the point. We cannot help thinking our author rather too fastidious in respect to the private baths. The ante-rooms and every thing connected with them, are infinitely more tidy and clean than at his favourite "Furstenbad" at Wildbad.

The font for drinking the water is certainly not quite like that at Marienbad or the Pump-room at Bath; while the very old dame who hangs over the slender rill, would seem a good specimen of Buxton longevity—perhaps of the salutary influence of the waters. It has neither colour, taste, nor smell. Its medicinal effects are much less determinate when taken internally. But, in either case, we imagine that these effects are better ascertained by experience on the spot, than by any hypothetical theory founded on caloricity, vitality, telluricity, &c. by temporary sojourners at this or any other spa. Sir C. Scudamore, assisted by Mr. Garden, detected 4½ cubic inches of azotic gas in a gallon of these waters, and to this Sir Charles attaches considerable importance.

"With regard to the Buxton water, (says Sir Charles) the subject of my present enquiry, it certainly happens that, simple as it appears in composition, it does prove inconveniently stimulating to some persons of full habit and of the sanguineous temperament. They complain of flushing, headache, and slight giddiness, and are deterred, by such symptoms, from proceeding in the course of drinking it. Many instances have come under my observation in which the exciting power of the water has been proved in the gouty patient; symptoms of a paroxysm having occurred in a few days after its commencement; subsiding, also, upon its being discontinued, and from the aid of medicine.

Others, and those especially who have a weakened condition of the nervous energy and of the muscular power of the stomach, complain that the water is felt as a dead weight on the stomach, that it is slow in passing off, and that, until it does so, they are much oppressed and inconvenienced.

These, however, are the exceptions, and not the rule; for, in general, the water agrees remarkably well, and is drunk freely without any unpleasant result; but, on the contrary, with benefit and satisfaction."*

The operation of these waters on the bowels is variously stated by different patients and practitioners:—some of the former representing it as aperient, others as constipating. The probability is, that Buxton water has no specific action on the bowels, in either way. There is no doubt, however, of its diuretic properties—and the author just named, considers it useful in many cases of indigestion where there is no inflammatory or congestive condition present.

* Second Edition, p. 22.

Sir Charles details the mode of taking the waters internally, and ascribes all their medicinal effects, thus taken, to the azote which they contain.

"For the same reason that many persons speak of this water as being too simple in its composition, too much like common water to claim any reliance upon it as a medicine, do I think favourably of its qualities; namely, from its purity, owing to the remarkably small proportion of its solid ingredients, and from its gaseous impregnation with azote, qualifying it in so eminent a degree to fulfil the valuable purposes of a stimulating diluent; and which, taken on an empty stomach, or scarcely occupied with any food, acts favourably, in the first instance, on this delicate organ, and quickly finds its way into the circulation, producing ulterior good effects. In diluting, and assisting towards the removal of any acrimonious secretions which may be delayed in the stomach and duodenum, this aqueous diluent may have much more useful effect than may be at first sight imagined; particularly by those who see a remedy only in what is very potent in its nature and composition."* 24.

It is as a bath, however, that Buxton has attained and still sustains its reputation. Sir Charles Scudamore is far from wishing that the subterranean boiler had thrown up water at a higher temperature than 82°—"so happily intermediate between the warm and cold bath," which neither excites by too much stimulus of heat, nor depresses by too much sedative influence of cold. In this he disagrees, *toto cœlo,* from Dr. Granville.

Dr. Robertson, one of the resident physicians at Buxton, remarks that—"chiefly owing to the alkaline properties of the water, the skin is speedily cleared of all scurfiness and impurities, and is rendered most singularly and delightfully smooth."

It has been seen that Dr. Granville experienced the very opposite effects. The doctor's sensibility was probably a little deranged at the time. It clearly was so at Wildbad.

"The mode of entering the bath is a point of some importance. It is necessary to make the immersion as quickly as possible, in order that the shock and the consequent re-action may be instantaneous. When there is no infirmity to prevent it, the patient should, with the least delay after descending the steps, fall forward, so as to receive entire immersion; and, at the first time, be contented with this, leaving the bath immediately. The stay in the bath is to be gradually increased afterwards, and the maximum duration will vary in different cases; this averages from four to fifteen minutes.

It is the fault of many to remain too long; in doing which, they are deceived in expecting the tonic effects of the bath. Those who are vigorous enough, and become accustomed to the bath, may make their plunge from the side of the bath, taking care, as I have known some very imprudently do, not to dive as it were with the head downwards. The gravitation of the blood in this mode might create serious mischief."† 34.

Sir Charles considers an hour or two before dinner as the best time of day for taking the bath, some exercise having been previously used. It is absolutely necessary that the bowels should be in a clear and open state, before the bath is commenced.

---

* Scudamore, 2d Edition. † Ibid.

"The class of patients (says Sir Charles) resorting to the Buxton bath comprise, for the most part, those who have suffered from either gout or rheumatism. But it is by no means equally proper for the gouty and the rheumatic invalid under circumstances apparently similar. The pains of chronic gout imply a state of the system which requires particular treatment by medicine, before the use of the bath should be considered admissible. This may often be necessary also in chronic rheumatism, but the condition is not so positive.

The bathing will be a valuable remedy to relieve that debility of limbs, and of the whole constitution, which is a frequent sequel to chronic gout, and which seems to partake very much of the nature of rheumatism. But as there is no such thing as a universal remedy, so it will not unfrequently happen that, when the frame has become enfeebled by the often repeated attacks of gout, the reaction in the natural bath is insufficient, and the immersion is followed by chilliness, nervous depression, and languor, and discomfort, throughout the day. It is obvious that in these instances the warm bath must be chosen, so graduated, that either the patient may be brought back to the use of the natural bath, or continue the artificial, in order that it may act as a tonic to the body, and not as a relaxant.

Till about twenty-five years ago, the warm baths had not been introduced at Buxton, and therefore it was not a matter of choice whether they should have the preference. But now it is a general rule, and one I think pursued with great propriety, that from one to three or four warm baths should be used as a preliminary to the natural bath; if one only, the temperature may be 95°; if in a series of three or four bathings, the first may be 95° or 96°, and the succeeding a degree less each time, the stay in the bath being also proportionably shortened. During the immersion it is useful to employ diligently a good flesh-brush all over the body, but especially over the most affected parts." 36.

In respect to rheumatism, if it partake of the acute character, Sir Charles prohibits the natural bath—which he has often known to produce acute rheumatism, when employed too soon during convalescence from rheumatic fever. Sir Charles very properly recommends that all inflammatory symptoms be removed before any bath be used. The following quotation is long; but it conveys important information to those practitioners at a distance, who are preparing to send their patients to Buxton.

"The state of the gouty patient also requires to be well considered before permission is given for the use of the natural bath. If the diathesis of gout be at all considerable, the production of more or less of a paroxysm would be a sure consequence from a few plunges; and although no serious mischief might occur from this result, yet disappointment would be experienced, and the loss of time would be regretted. In all doubtful cases, the tepid bath should alone be the chosen remedy; and, in conjunction with it, such medical treatment as may prepare the constitution favourably for the best effects of the natural bath.

Some persons having suffered much misery and discomfort from various obscure symptoms, which commonly receive the name of suppressed gout, have resorted to Buxton with a view to elicit the disease in a definite form, and fix its action. For them, the warm bath is obviously the remedy; and indeed in cases of this nature, which are rare amongst the visitors of Buxton, it is right to say that, for the most part, a still more appropriate remedy might probably be found in the waters and baths at Bath; which, although not so fashionably resorted to as half a century or more ago, cannot have lost any part of their admirable medical powers.

Even when the gouty patient does appear in a fit state to use the natural bath, it will sometimes happen that, in a short time from its commencement, an attack of gout will unexpectedly take place; yet I have rarely seen it happen severely, and never really to be injurious. It is desirable that it should be brought out rather than lurk in the system; and when such paroxysm has been treated judiciously by medicines appropriate to the gout, the use of the bath may be resumed, and will most probably be quite successful.

Both gout and rheumatism affect various textures of the body, as the synovial membranes, those immediate to the joints, and the bursæ mucosæ and the sheaths of tendons; the aponeurosis or tendinous expansions of muscles, tendons, ligaments, and nerves; and cellular membrane rather secondarily than primarily. Sometimes, yet very rarely, the belly of a muscle is affected by rheumatism, being swollen and tender; far more commonly, the seat of the disease is in its expansion into tendon, and its tendinous insertion. In general, the synovial textures and the ligaments are affected in common; occasionally, much more in the one kind of structure than the other, but seldom exclusively. The Buxton bath will be a very proper remedy for trial in all these morbid states, but it will not prove equally efficacious in all.

In rheumatism of the ligaments, its remedial powers are the most quickly shown; and, next, when the disorder affects the bursæ. The cases most unyielding, are old affections of the sciatic nerves; yet I can state, with truth, that I have had the satisfaction of witnessing very great relief afforded by the bath in some chronic cases of sciatica of very long standing, and which had resisted the ordinary means of treatment.

Neuralgic disease embraces a very important class of cases. Its most frequent form is neuralgia rheumatica, or that painful state of nerves, acute or chronic, which has evidently been induced, like ordinary rheumatism, by exposure to wet and cold, when the nervous system has been in a morbidly susceptible state. Most frequently it appears as sciatica; but any other of the nerves of the extremities may be similarly affected. The branches of the femoral nerve are not unfrequently in this way disordered; occasionally, those of the brachial nerve, and other muscular and subcutaneous branches.

The bath and the use of the pump will be proper in all cases of this description, when the nervous painful irritation is not attended with inflammatory action; or with some evident disorder of the internal functions, requiring appropriate medical treatment as preparatory to the bath.

Neuralgia spasmodica, or tic douloureux, cannot be treated specifically by the bath; nor will it be proper in this severely distressing complaint, except as an occasional refreshment and source of invigoration, and provided that there are no contra-indications for its use. It can only be recommended as an adjunct to other treatment, and under great regulation, and medical guidance.

In that infirm state of the lower limbs which almost amounts to complete paralysis, and which is always to be referred to a disease in the spinal column, the use of the bath and of the pump is admissible, and I have seen it prove useful in such cases; but we must not expect more than slight benefit; and indeed all other means that can be devised are too commonly of little avail towards a cure. Great alleviation, however, of suffering and inconvenience may be rendered to the afflicted patient. It is not the reproach of the medical art, that every case of disease does not allow the possibility of cure.

A very valuable part of the Buxton treatment consists in the use of the forcing pump, which is a remedy of considerable power; and being so, its administration requires more exercise of discretion than I think it commonly receives. Some persons think they cannot have too much of it, either as to its strength, or duration, or repetition; and again, and again, have I seen inflammatory irritation produced by the violence of its employment.

It is particularly useful in reducing enlargements of the bursæ, and in restoring tone to weakened ligaments.

It is always proper to commence the use of the pump with moderation; for example, from ten to twenty strokes on the particular part affected, and to be moderately given. According to the effects produced, it is to be increased, both in the force with which it is applied, and in the number of the strokes. When the patient, throughout the day afterwards, feels more or less sensible benefit, and does not experience nervous irritation unusually after getting to bed, it may be received as a proof with regard to the pump, as to the bath, that the treatment agrees, and that it has been correctly used.

In particular cases, it may be desirable to use the pump on the days of omitting the bath.

I am convinced that the advantages of the Buxton bath are most materially increased, when proper friction and *shampooing** are used in conjunction with it. I think the early part of the day is the fittest time; although, in some cases, it is not very material when it is used. When the circulation in the lower extremities is languid, and there is a disposition to œdema, this treatment would be very suitable soon after the quitting the bath; provided that the invalid is not fatigued, and requiring rather some repose on the sofa than further excitement of any kind.

By such treatment the circulation of blood in the weak muscles is actively promoted, without the least fatigue to the patient; and other good effects upon the infirm limbs are by degrees produced. I may briefly define the advantages of this treatment to consist in the influence which it may possess, to relieve the parts from the effects of preceding effusion by exciting the absorbents to unload the cellular membrane; to assist in restoring the lost freedom of motion in the tendons and ligaments; to renovate the capability of proper contraction and relaxation in the muscular fibres; to improve the circulation as above stated; and conduce to a more perfect transmission of the nervous influence.

It will be asked in what other kinds of disorder, besides gout and rheumatism, may the Buxton bath be looked upon as a remedy.

Since so much can be justly said of its restorative tonic powers, it would naturally occur to the minds of most persons, that paralysis would be a very fit occasion for its useful influence; the natural association being, in the view taken of the question, debility of nerves and muscles, and a soothing tonic bath. But there is no kind of disease more requiring cautious consideration in the treatment. It is true that many cases of paralysis owe their origin to some disordered condition of the spinal marrow; but, in most cases, and especially the recent, there is prevailing an attendant ready susceptibility in the vessels of the brain to be unfavorably excited; so that if the general circulation be over stimulated by errors in diet, strong liquors, or disturbed in its balance by the influence of a plunging bath, of any temperature, causing a re-action, and a consequent forcing of the circulation, if I may so express it, apoplexy, or a condition bordering upon it, might be produced; and, indeed, I have witnessed, more than once, such a result to happen from the imprudent use of the Buxton natural bath.

In old cases of paralysis of the limbs, which, from their origin, have been clearly referrible to the spinal marrow, and not to the brain, the use of the natural bath and of the pump is very admissible, and I have known such patients use both with perfect agreement; yet cases of this unfortunate description so little admit of curative treatment, that any great benefit is not to be expected.

In every case of paralysis arising from disorder of the brain, it must be highly improper to make use of a plunging bath; nor should a hot-bath of high temperature be permitted, for the obvious reason of its being dangerous to excite the circulation in a sudden and great degree.

---

* "There are, at Buxton, appointed persons always attending in the season, well qualified by their experience to render all the benefits which can be derived from this auxiliary mode of treatment."

In cases of this nature, it is very difficult to do good by any active medical interposition, but very easy indeed to do harm; and a prudent physician is therefore more inclined to use passive than active measures; to employ, chiefly, *la medécine expectante*, and to lay down rules of general management in the diet, exercise, conduct of the mind, and every moral circumstance which can be under the control of the patient himself, or his friends.

In cases of paralytic weakness of a limb, the consequence of gout, rheumatism, the introduction of lead into the system, inveterate disorder of the digestive organs, and bodily accident, the use of the natural bath and the pump will be unquestionably proper :—I may add also, for the effects of strains.

It is very common for persons in health, staying at Buxton, to make use of the natural bath as a matter of enjoyment; and, so that it is used with discreet frequency, and under proper regulations, I see no objection to it; nor am I aware of having seen any ill consequence resulting from it, where the prudence for which I stipulate has been observed. I must here, however, remark that it will not invariably agree with persons who call themselves well; so true is it that, from idiosyncrasy of constitution, there are individuals who find rather disadvantage than benefit from persisting in the use of any kind of bath whatever." 42.

The Buxton season lasts from the middle of May till the end of October. Numerous cases are detailed by Sir Charles illustrative of the efficacy of Buxton waters, at which hygeian font he has now presided for very many years, during the Summer and Autumn. These cases we shall pass over—as well as the observations of Dr. Granville, who, like ourselves, have only *distal* experience of the waters in question. The following passage, however, deserves a comment.

" Where I have seen the Buxton waters perform wonders has been in persons who, having had annual, or perhaps semi-annual attacks of genuine painful gout, have not courage enough to support pain, and fly at once to that *curse of the human constitution colchicum*, to quell the knawing dogs, and purchase a lull from sufferings at the *inevitable* risk of multiplying the attacks of the disease."

From this it appears that Dr. Granville leaves painful attacks of gout to the treatment of Dame Nature! It is clear that Dr. G. never had gout himself—and we must say that the mode of treatment at which he hints is not only injudicious, but it is positively *injurious*, and tends to multiply the attacks of the malady, and also the sequelæ of the disease. There is not a safer medicine in the Pharmacopœia than colchicum properly administered. Its assistance in removing the attack, never augments the tendency to return, when it is conjoined with proper remedies and followed by proper regimen. Upon second consideration, we shall introduce the following case from Sir Charles Scudamore, as it will shew the bad effects of using the waters of Buxton without due preparation by medicine.

" A gentleman, aged 50, of the nervous temperament, formerly a free liver, had suffered long and severely from acute gout, but latterly from the chronic form of the complaint. He was reduced in flesh and weak. He was affected with alternating pains in the head and limbs, so much increased by change of weather that he considered them to be rheumatic, and under this impression visited Buxton, for the purpose of using the natural bath. Neglecting all preparatory treatment, he bathed three times, within five days, in the public bath. On each occasion he felt chilled at the time of immersion, and did not receive comfortable warmth afterwards. His head was constantly painful, and the limbs in no degree relieved. On the day following the last bathing, I was consulted, and found him suffering from many urgent symptoms. He described his head to feel as if too full of blood, and he had great confusion of thought. Gout had fixed in one foot and one knee, with much pain, but only slight signs of inflammation. He had general pains over the body, with frequent nervous shiverings. I found the strongest indications of error in the digestive organs, *and prescribed active aperients and alteratives, in conjunction with the moderate use of the acetum colchici.* Leeches were applied to the temples, and a very small blister to the neck.

By these means all the active symptoms of complaint were in a short time removed, and I then directed the use of the warm bath, beginning at the temperature 96°, and gradually reducing it to 90°; after which he returned to the natural bath with perfect success. He continued it for six weeks, under strict regulation, and obtained a very satisfactory recovery. He also derived benefit from drinking the water of St. Anne's well." 45.

### 4. Leamington.

Leamington is a place of many medicinal springs; but the main spring of the place is a medicinal doctor. Physicians, like other people, sometimes build houses, but few, if any, have ever founded cities or towns. The father of physic, indeed, enriched his native isle by attracting strangers thither for the benefit of his advice, but the Coan sage constructed no town out of the profits of his profession. Virgil is said to have written his own epitaph, part of which ran thus: "Mantua me genit—Roma me fecit." If the far-famed Hippocrates of Warwickshire were to commence his epitaph with "Leamington me genit," the grateful town, had it as many tongues as it has tiles, would instantly respond—" Jephson me fecit," and thus interrupt the epitaph writer. But, however the final inscription may run, or whatever the doctor's contemporaries may think or say, it seems unquestionable that he must have possessed no mean degree of skill and talent, who could convert grains of salt and steel into tons of gold and silver.

The medicinal springs of Leamington all lie within the range of a musket-shot from the bridge over the Leam. And yet their names have undergone such changes within a few years, that it took us a whole morning to decypher Dr. Loudon's account of them, published in 1831. The worthy and talented doctor enumerated them according to their ages, but fortunately annexed their localities, which enables the stranger to recognize them in despite of the changes in their titles. Passing over the little bridge that spans the almost stagnant Leam, and leaving two great spas on our right hand, we come to the "Old Well," or No. 1, the father of them all, and whose origin is shrouded in the mists of antiquity. It is in front of an old church, near the river, in Bath Street, and has two spouts or pumps—one on the outside, *pro bono publico,* the other inside of a little pump-room, for all who can afford to pay, either by the day, week, or month.

The water of this well presents (in the pint) the following ingredients:—

| | |
|---|---|
| Sulphate of soda .................. | $40\frac{1}{3}$ grs. |
| Muriate of soda................... | 40 |
| Muriate of lime ................... | $20\frac{1}{2}$ |
| Muriate of magnesia.............. | $3\frac{1}{4}$ |
| Peroxide of iron............a trace. | |
| And the same of iodine and bromine. | ——— |
| Total.. | 105 grs. |

This pint also contains a small quantity of oxygen, half a cubic inch of azote, two cubic inches of carbonic acid gas, but no sulphuretted hydrogen.

No. 2.—Formerly ABBOTT'S—now GOOLD'S.

This was discovered in 1784, and is situated a little farther on, on the opposite side of the same street, and to the south of the Bath Hotel. It is called "Goold's original Spa or Baths." When first sunk, it caused the water in its ancient neighbour (the Old Well) to subside several feet. Its contents, as might be expected, are almost exactly the same as No. 1, except that it presents five or six grains more of common salt. Here the first baths were erected in Leamington, and are now in fair and respectable condition. It has a large Turkish bath, in addition to cold, warm, vapour, and shower-baths.

No. 3.—WISE'S, now CURTIS'S WELL.

This was sunk in 1790, and is situated at the corner of Bath Street and the Royal Parade. Its analysis, and that of the other two wells, was published by Dr. Lamb, then residing in Warwick, and tended to bring Leamington into more repute, though still local. Its constituents differ only from those of the others, in shewing less muriate of soda, by twenty grains—and more muriate of magnesia by the same quantity.

No. 4.—ROBBIN'S WELL, now THE VICTORIA.

This was opened in 1804, and is one of the two grand pump-rooms on the banks of the Leam, and close to the bridge, but on the south side of it. It was now that a revolution took place in the spas of Leamington. From being drunk at the original fonts, and as they issued from their subterranean reservoirs, they were then enclosed in buildings—pumps inserted into the natural wells or fountains—the medicated streams foamed from silver cocks—and were distributed to invalids by the hands of fair priestesses of the respective temples of Hygeia.*

As the reputation of the springs rose, so did the number of houses and hotels. Two years after the opening of the VICTORIA, two other springs in the same locality—

Nos. 5 and 6,

READ'S, now LEE'S WELLS—sprang into existence—one a strong sulphureous—a second Harrogate water—the other a saline chalybeate.

* This well contains ten or twelve grains less of sulphate of soda (viz. 28 grains in the pint) than the original well, but presents no other differences worth notice.

ANALYSIS.

The SULPHUREOUS spring shews better than three cubic inches of carbonic acid gas—and more than an inch of sulphuretted hydrogen gas in the pint. It contains 28 grains of sulphate of soda—25 muriate of soda 15 muriate of lime—9 muriate of magnesia—in all 79 grains in the pint. Its taste is not quite so nauseous as that of Harrogate; but it is much used both as baths and for drinking.

The SALINE neighbour shews 103½ grains of solid matters in the pint, of which 30½ are sulphate of soda—43 muriate of soda—18 muriate of lime—10 muriate of magnesia—⅓ of a grain per-oxide of iron. Its taste is unequivocally chalybeate.

Hitherto the medicinal springs were all confined to the Old Town, on the south side of the Leam. In 1808, a spring was found on the north bank, exactly opposite the Victoria, to which the name of

No. 7—or "ROYAL SPA"

was given, and over it was erected an elegant pump-room, with extensive suites of baths—and a large piece of ground for promenades, music, &c. This spa also presents two springs—one sulphureous (weak), and the other saline. The SULPHUREOUS only contains fifteen grains of solid matters in the pint, of which six are sulphate of soda—five muriate of soda—three muriate of lime. The sulphuretted hydrogen gas amounts to one cubic inch and a fraction. The taste, or rather the flavour, is, however, decidedly sulphureous—the taste very weak.

The SALINE Chalybeate Well here is the strongest in Leamington, affording 136 grains of solid matters in the pint. Of these, thirty-two are sulphate of soda—sixty-seven muriate of soda—twenty muriate of lime—twelve muriate of magnesia—one grain nearly of peroxide of iron —three cubic inches of carbonic acid gas. This is the spa that appears to attract the greatest number of drinkers—partly from the strength of the saline chalybeate—partly, from the size of the pump-room—and partly for the pleasure-grounds and music.

But it was in 1819, that the good fortune of Leamington was supposed to have been consummated by the discovery of three springs in Clemens Street, close to the Royal Parade—one chalybeate—one pure saline—and one sulphureous. The chalybeate was found to contain the enormous quantity of eight and-a-half grains of peroxide of iron in the pint, held in solution by silica!!! The imperial gallon of this water contained 1,085½ grains of solid matters—of which 274 were sulphate of soda—442 muriate of soda—200 muriate of lime—31 muriate of magnesia—silica 68—peroxide of iron 68 grains!

We anxiously directed our steps to this wonderful spring; but what was our astonishment to find it closed up—as we were informed, for two or three years past. No one could tell us how or why. The "IMPERIAL FOUNT," as it was termed, and as is still written up on the house—had vanished into air, or gone back to its hidden source in the earth! After puzzling our brains for a good while, the solution of the enigma suddenly burst on our mind. It was Dr. Jephson's powerful MAGNET that had drawn the steel out of the Imperial Fount—and hence the "MAGNETIC IRON" which he has so freely prescribed for many years past.

The alteration which even ten or a dozen of years have produced in Leamington, is absolutely astonishing! A new town—JEPHSTOWN, it ought to be called—has spread up from the lazy Leam, in parades, crescents, and elegant streets, till they have enclosed the mansion of their architect, which used to stand alone in its glory. The place, however, is overbuilt—and for this good reason, that its prosperity and increase of visitors do not rest so much on the efficacy of its waters as on the reputation of its MAGNUS APOLLO. Let the SUN withdraw his beams, and Leamington will experience a sad falling off!

Still, it must be acknowledged that the waters of Leamington are powerfully remedial agents, and are formidable rivals of those at the far-famed Cheltenham. They may be taken with perfect safety at all seasons of the year, though the most usual period is from the beginning of May till the end of October—on account of the auxiliaries which air and exercise bring to the spas. The best time for taking the waters is before breakfast—and next to that about noon.

### THE SALINE SPRINGS.

Some preparatory steps ought to be taken, both in respect to aperient medicine, and the reduction of vascular fullness when any such exists.

"The preparatory steps being premised, a common pint of the water may be taken; one half about seven o'clock in the morning, on an empty stomach; the other in about twenty minutes afterwards; walking exercise being used between the first and second part of the dose, and after both have been taken. For children of twelve years of age, one-half of this quantity will be sufficient; for those of six, one-fourth. Under the age of six, they should scarcely ever be employed.

The immediate effects of the saline waters, when taken internally, in aperient doses, are of three kinds. Either they produce an increased action of the kidneys, or bring on nausea, sickness, headache, flushings of the face, distention of the stomach, determination to the head, and other disagreeable symptoms; or, finally and most frequently, they act on the bowels without inducing any of these signs. In the first case, they require only to be increased in the dose, to produce the aperient effect; while, in the second case, their use is not prohibited by the unfavourable effects arising from their employment, unless the remedies resorted to for removing them should prove ineffectual. The second class of symptoms frequently supervene from a deranged state of the alimentary canal; and by a little attention to the digestive organs, and to the dissipation of the gases, may be avoided. When they pass off by the kidneys, their use may always be regarded as pretty safe, and their action as salutary."—*Loudon*, p. 52.

The saline waters of Leamington are most frequently resorted to for affections of the digestive organs, and where aperient effects are desired and desirable. In several external diseases, as chronic ophthalmia—ulcerations—and some cutaneous complaints, for which, however, the sulphureous waters are more adapted.

"The saline waters of Leamington are also entitled to great regard, as alterative agents; by which is meant, a class of substances that possess the power of gradually improving the condition of the system, without affecting the patient very sensibly at the time they are taken. The minute division of the ingredients affording a very easy entrance for the particles into the vascular system, necessarily renders their influence very extensive over every tissue of which the animal frame is composed; and that this absorbent action does take place there can be little doubt, from the diuretic power which they possess. It is thus, chiefly, that the saline waters are so much celebrated for those disorders, which, in their first and most inflammatory state, affect the whole system; and which, afterwards, leave a weakness and loss of motion, with transient pains, and other adynamic symptoms in the limbs. In these affections, however, of which gout and rheumatism may be adduced as examples, no patient should venture on the internal use of the class of remedies under consideration, until every discernible sign of the active state of the gouty and rheumatic diathesis shall have completely subsided. Nor should a slight exacerbation of the disease, more especially of the gout, induce the patient to abandon the use of the water. Such a consequence is an occasional occurrence, and it has, with some propriety, been referred to the stimulating properties of the muriates. It will be prudent, therefore, to suspend the use of the springs during the attack, and to resume them at some after period, when these symptoms have passed away."—*Loudon*, p. 55.

Dr. Loudon considers these saline waters as extremely useful in cachexy — strumous swellings — abdominal tumefactions — mesenteric disease—white swellings—spinal affections, &c. not from any specific powers or qualities of the waters, but from their alterative effects and improvement of the general health.

" Mild as the saline waters usually are, an indiscriminate use of them, like the abuse of every other medicine, proves very hurtful to the constitution. When repeated too often, a febrile state is induced by the application of the saline particles to the mucous membrane of the intestine, which, by withdrawing at the same time a quantity of fluid from the general circulating mass, is followed by a diminution of the vital functions, an effect which, it is evident, in enfeebled constitutions, it is of the greatest importance to avoid. Not less frequently do the harassing and painful symptoms of hemorrhoids follow the immoderate use of the waters. When employed in drastic doses, diarrhœas, of the most troublesome nature, frequently supervene; more especially in habits of a peculiarly irritable nature."—*Loudon* p. 57.

### Sulphur Springs.

These waters are taken in the same doses, and in the same manner as the salines; but as the taste is not very agreeable, some peppermint or cinnamon water is often used with them. The palate, however, soon gets reconciled to them.

" When the sulphureous waters are likely to be serviceable, they excite, immediately after they are taken, no very particular sensation of any kind. On the other hand, when they sit unpleasantly on the stomach, occasion head-ache, dryness of the tongue and fauces; or sickness, and do not pass off by perspiration, or excite some of the excretions, their operation may be looked upon as unfavourable; and they ought, after the auxiliary means have been fairly tried, to be discontinued."—*Loudon*, p. 59.

Dr. Loudon advises that heat should hardly ever be applied to the sulphur waters, as the gases are thereby dissipated, and much of their virtues lost. When they are found to be too strong for the stomach, some hot saline water should be added, and the mixture drunk immediately off. We recommend the following passage to the attention of our *Anglo-Germanic* spa doctors who are horror stricken at the idea of any medicinal auxiliary to a mineral water. The fact is, that most of these pharmaco-phobists know extremely little of the practice of medicine.

"In almost every case for which the sulphureous wells are resorted to, the preparatory plan, pointed out for the saline water, is necessary. In order, also, to produce the full effect, it will be proper, in numerous complaints, to assist the waters by some medicine calculated to act as an adjuvant during the whole period of the course. Not unfrequently it happens, with regard to the sulphureous waters, as well as the others, that, instead of the aperient effect which they are all primarily calculated to produce, there is, simply, a fluid discharge from the intestines; which, by being deceitful to the patient himself, at the same time leaves the cause of the disease lurking in the constitution. If the object be to evacuate completely the contents of the alimentary canal, recourse is frequently had to a quantity of the prepared salts.* But, valuable as these are as laxatives to obtain a free expulsion of the contents of the bowels, some more active aperient medicine must be substituted. Nor should it be forgotten, that in every ailment there is usually more than one indication to fulfil, towards effecting the recovery of the patient; and hence, in a variety of cases arises the advantage of combining a suitable auxiliary treatment along with the water, by means of which the disease may be cut short in a much less period of time than if the mineral fluid was simply employed."—*Loudon*, p. 61.

An alternation of the sulphureous, with the saline or chalybeate waters is recommended by Dr. Loudon. In cutaneous complaints, these waters require to be continued, both internally and externally, much longer than the others.

"The sulphureous waters are contra-indicated in acne rosacea and punctata. When employed as purgatives, they are also, in large doses, improper in old people with leuco-phlegmatic habits; for, as in the previous kind of water, by diminishing the quantity of fluids from the circulating mass, emaciation, low spirits, general debility and dropsy, may succeed. In very large doses they produce emesis similarly to the saline and the chalybeate."—*Loudon*, p. 64.

### CHALYBEATE SPRING.

Dr. Loudon makes many judicious observations on the use and abuse of the Chalybeate Spring at Leamington; but as this well is shut up, and as we have introduced sufficient remarks on the action of chalybeates already in this article, we shall pass over this chapter, with the exception of the following short passage.

"Although, generally speaking, before commencing the saline and sulphureous waters, it is advisable, in the greater number of cases, that the bowels should be regulated by some suitable medicine, this preparatory step is more particularly called for previous to a course of the chalybeate water. The astringent effect which the iron spring possesses, is apt, now and then, to produce a much less aperient action on the bowels, than the composition of the spring would lead to infer; and hence, for the same reason, it is necessary to continue some aperient medicine during the whole course of the water."—*Loudon*, p. 65.

---

* "The salts, however, dissolved in water, are an excellent substitute for the springs. They are prepared at Mr. Smith's Establishment, in Bath-street, and sold by him, and at the different Chemists in Leamington; and in London, at Messrs. Barclay and Son's, Fleet-market."

Leamington, as we before observed, is well supplied with bathing establishments of every description. It is a quiet—we had almost said a very dull place: but the vicinity, especially Kennilworth, Warwick Castle, &c. afford agreeable and interesting excursions. Leamington is an aristocratic watering-place, and, as such, will be preferred by that class of society, to Cheltenham, which, like Brighton, has become strongly tinctured with democracy.

"The importance of Leamington, as a watering-place, may be inferred from the extreme rapidity with which it has risen from obscurity to be a town of such considerable magnitude. This has, with some degree of justice, been attributed to the number, variety, and abundance of its mineral streams. Having eleven different wells, and uniting, in a single spot, waters similar to those of Harrogate, Tunbridge and Cheltenham, the sick are neither necessitated to wander about from place to place, seeking that which is most applicable to the complaint under which they labour, nor obliged to add foreign ingredients to increase their powers."—*Loudon,* p. 86.

Considering, however, that Dr. Loudon's favourite spring, the Strong Chalybeate, is shut up, and that, at Harrogate there are two, and at Cheltenham one very good chalybeate, the exclusive advantages of Leamington cannot, without partiality, be maintained.

### 5. Cheltenham.

Who is not as familiar with the name of Cheltenham, as with that of the street in which he resides? What man, woman, or child, in Great Britain or in any of her colonies, has not heard of No. 4? What planter in the Antilles, who had ever chewed a sugar-cane, flogged a nigger, or swilled sangaree, has not repaired to "No. 4," in the hope of washing out the bile from his liver, and all record of Wilberforce from his memory? What Nabob of the East, who had eaten curry till his skin was as yellow as a star pagoda, did not ply "No. 4," on his return, in order to expel the mulligatawney from his complexion? What alderman who had gobbled green fat and callipee till his abdomen outmeasured a huge turtle, does not "clear out," at "No. 4," preparatory to the re-shipment of a new cargo? In fine, every individual, old or young, in this country or our dependencies, who keeps an eye to No. one, must keep the other eye on "No. 4," at Cheltenham,—if he mean to live long, feed well, and sleep soundly. And yet "No. 4" is nearly *no more!* A new Naiad has opened a shop within a few inches of the old Hygeian Goddess, and entitles herself "No. 4. a." running away with three-fourths of the custom from her ancient neighbour. This new aspirant will be noticed presently.

Cheltenham, in our opinion, is one of the cleanest, most cheerful, and handsome provincial towns in England, or in Europe. It is sheltered and nearly surrounded by moderate hils, cultivated to their summits, and presenting a most picturesque panorama from the cupola of the Pittville Pump-room. It has been bruited that the reputation of the waters has declined. The rapid increase of the population from 23 thousand, in 1831—to 35 thousand in 1841, would seem to negative this report.

"The principal thoroughfare from the High-street is the Colonnade, which leads to one of the most beautiful promenades in Europe, at the extremity of which is the Queen's Hotel, a majestic building, and, as an establishment, stands without a rival. On this spot formerly stood the Sherborne Spa. Around this neighbourhood are some of the most lovely and delightful rides and walks in the kingdom. These embowering shades and pendant woods, form an enchanting retreat during the Summer months, and are the general resort of all the rank, beauty, and fashion of Cheltenham, where

'From Courts and Senates each may find repose.'

The Promenade Terrace is much admired for its elegant and classic design. An opening from the Terrace conducts the Visitor to the entrance of the Old Wells, as well as to the Bay's Hill Estate. The stately avenue of Elms, planted in 1739, and now in the pride of luxuriant foliage, are here seen to the greatest advantage. They are justly the theme of admiration; and contribute, in no small degree, to the beauty of the landscape. It is in visiting such enchanting scenes as these that

'The smiling God is seen; while water, earth,
And air attest His bounty.'

The streets, terraces, crescents, villas, and parks are now rapidly stretching away towards the Cotsdown Hills, which they will probably one day climb, in pursuit of cool air.

The Springs of Cheltenham are numerous; but they are easily traced—not indeed to their sources, but to their exits in the various pump-rooms. Many acres of the rising grounds about Lansdowne Crescent are *mined*, as it were, and the waters conveyed for more than a mile by subterranean conduits to the Montpellier Rotunda and the Laboratory of Mr. Thompson, for the preparation of Cheltenham Salts. Into some of these medicinal mines we descended, and saw the genuine waters gushing into the wells at a depth of 40 to 80 or 100 feet, and thence gliding away to their respective destinations, for the benefit of mankind. How different the results of these hygeian mines from those which disgorge their gold and silver for the corruption of the human race!

"Effodiuntur opes irritamenta malorum."

But we must glance at the Spas themselves.

### THE OLD WELL.

This ancient spa, (at which our good old GEORGE the THIRD quaffed,) together with its magnificent avenue of elms. is sorely eclipsed by its more modern neighbour, the MONTPELLIER, and nearly left alone in all its glory! The contents of a pint are as follows:—

| | | |
|---|---|---|
| Muriate of soda | 58·20 | grs. |
| Muriate of lime | 6·21 | |
| Muriate of magnesia | 2·54 | |
| Sulphate of soda | 14·56 | |
| Total | 81·51 | |

There is a very small proportion of carbonate of iron in this well. At this spa there is also a

"Strong Chalybeate Saline."

| | |
|---|---|
| Muriate of soda.................... | 17·60 |
| Muriate of lime.................... | 3· |
| Muriate of magnesia................ | 3·30 |
| Sulphate of soda................... | 43·20 |
| Carbonate of iron a large quantity. | |
| Total.... | 67·18 |

The Old "No. 4" of the Old Well contains;—

| | |
|---|---|
| Muriate of soda.................... | 47· |
| Muriate of lime.................... | 4· |
| Muriate of magnesia................ | 7·½ |
| Sulphate of soda................... | 59· |
| Total.... | 117·½ |

This also contains some iron. It is evidently a most valuable water, however eclipsed by its new neighbour.

The Old Wells also present sulphuretted salines, which need not be noticed.

Saline Chalybeate.—(No. 1. Montpellier.)

| | |
|---|---|
| Sulphate of soda .................... | 14·7 |
| Sulphate of lime .................... | 1·3 |
| Sulphate of magnesia ................ | 4·0 |
| Muriate of soda ..................... | 27·0 |
| Bicarb. of soda...................... | 1·1 |
| Oxide of iron ....................... | ⅓ of a grain. |
| Carbonic acid gas 2½ cubic inches. | |
| Total.... | 48·4 |

This is evidently an excellent saline chalybeate, since the aperient salts and muriate of soda must check the bad effects of iron, in inflammatory habits.

Sulphuretted Saline.—(No. 2, at the Montpellier.)

Gazeous Contents, 1·6 cubic inch of sulphuretted hydrogen.

SALINE CONTENTS.

| | |
|---|---|
| Muriate of soda ..................... | 35·3 |
| Sulphate of soda .................... | 28·4 |
| Sulphate of magnesia ................ | 7·2 |
| Sulphate of lime .................... | 3·1 |
| Oxide of iron ....................... | 0·4 |
| Hydriod. of soda..................... | 0·15 |
| Total.... | 74·57 |

The next saline (No. 3) at the Montpellier is merely a weaker one than No. 2.

No. 4, or PURE SALINE.—(Montpellier.)

This was long the lion of the spas at Cheltenham—and is still a very valuable water.

COMPOSITION.

One and four-tenths of a cubic inch of carbonic acid.

| | |
|---|---|
| Muriate of soda | 52·4 |
| Sulphate of magnesia | 14·2 |
| Sulphate of soda | 17·2 |
| Bicarb. soda | 1·2 |
| Sulphate of lime | 2·7 |
| Carbonate of lime and of magnesia | 1·1 |
| A trace of hydriodate of soda. | |
| Total.... | 88 |

No. 4, A., or the reigning favourite at the Montpellier Wells.
"IODURETTED SALINE."

COMPOSITION.

Carbonic acid 1·6 cub. inch. A trace of sulphuretted hydrogen.

| | |
|---|---|
| Muriate of soda | 51·4 |
| Muriate of lime | 8·3 |
| Muriate of magnesia | 7·3 |
| Sulphate of soda | 14·0 |
| Sulphate of magnesia | 17·1 |
| Sulphate of lime | 2·1 |
| Bicarb. soda | 2·4 |
| Carb. of lime and carbonate of magnesia | 3·2 |
| Hydriodate of soda | 0·25 |
| Total.... | 106·25 |

It will be seen that this water contains the same quantity of aperient salts (both sulphates and muriates) as No. 4, besides a larger quantity of lime, and 7½ grains of muriate of magnesia. The iodine, too, is in much larger quantity than in No. 4. It is not without reason, therefore, that it is more generally taken than any of the other waters here.

There are two other pumps here in the Rotunda, No. 5 and No. 6—the former called "THE CHALYBEATED SALINE," and contains 47 grains of sulphate of magnesia—ten of muriate of magnesia—and only nine of muriate of soda—with nearly half a grain of oxide of iron, with some iodine and bromine.

The other (No. 6) shews a large proportion of muriate of soda (59 grs.) with 4½ grs. of muriate of magnesia, and only twelve grains of sulphate of soda. It is called the "MURIATED SALINE."

There is also a "CHALYBEATE" at Thompson's Laboratory, containing one-third of a grain of oxide of iron in the pint, with various salines, and about half a cubic inch of sulphuretted hydrogen gas, which keeps the iron at the *minimum* of oxidation.

### Cambray Wells.

Not far from the Montpellier Gardens, at the southern end of Rodney Terrace, are two wells, called the "Cambray Wells"—one the "Original Chalybeate," containing nearly a grain of carbonate of iron in the pint, with about seven grains of saline matters—two grains of muriate of lime and magnesia—three of sulphate and muriate of soda—one of lime—and one of magnesia. This may, therefore, be considered as nearly a pure chalybeate, and shews three cubic inches of carbonic acid gas in the pint.

"The Pure Saline"—(Cambray,)

Contains 77 grains of saline in the pint, viz.—51 muriate of soda.
$8\frac{1}{2}$ muriate of lime.
17 sulph. sodæ.
A mere trace of iron.

### Pittville Waters.

Descending to the High Street from the Old Wells, Montpellier, and Cambray, we ascend on the opposite side of the town, full half a mile, to one of the most magnificent pump-rooms in Europe, with gardens, ponds, shrubberies, and a lofty dome, from which a splendid panorama bursts on the eye, bounded by the beautiful Cottswold Hills. Here are three springs—a strong and a weak saline, together with a sulphuretted saline. The latter has not been yet analyzed. Both of the former, however, are very weak. The stronger contains only 46 grains of saline matters in the pint—viz. sulphate of soda, 17 grains—muriate of soda, 27 grains—with hardly anything else.

The weak saline shews 7 grains of sulphuric acid—26 chlorine—17 sodium—3 soda—1 gr. lime.

Thus then we see that there is a great variety of medicinal waters at this place—enough indeed to answer almost every indication. We have heard that the waters are found to vary occasionally, in some of their sensible qualities, especially the taste; but we have no proof, or even presumption, that their chemical constituents undergo any material variation in consequence of time or season. Much less do we give any credence to the absurd and scandalous tale about cart-loads of Epsom salts being weekly imported for the supply of the springs, or rather of the laboratory for making the Cheltenham salts.

The last time we visited Cheltenham (Aug. 1841) we observed that a general impression prevailed there that Dr. Granville had depreciated the waters. We must candidly say that, a perusal of his work has not led us to any such conclusion. He avers, indeed, that the waters of Cheltenham will not cure all diseases—and that they are not proper in the acute or active stage of any disease. These are truths which will apply to any or all of the most celebrated waters of Germany, or any other country. Dr. Granville admits (a most remarkable admission on his part) that Medicines are not only useful, but absolutely necessary, in those diseases for the cure of which Cheltenham is renowned; and that in the good old days of his early practice, patients were only sent to Cheltenham when they were convalescent, and when the London or provincial doctor found that he could do no more.

"I would illustrate my position by a single example taken from what, in our days, may be considered as an almost every-day occurrence in medical practice. There was a time, say twenty years ago, when a patient labouring under extensive disease of the liver, commonly so called, and no matter how produced, whether by a residence in a tropical climate, or from sedentary life and anxiety of mind, or through frequent imprudence committed at the table, would first undergo that suitable treatment under a skilful physician, which sooner or later, and *alone*, does successfully overcome this class of disorders. But inasmuch as even the skilful physician and the most appropriate treatment could not do all in such cases; and as after having cancelled the positive disease by remedies, the medical attendant often found it difficult, if not impossible, to restore by the ordinary means the constitution to its normal state—a thing only to be obtained through the agency of such chemical combinations as were found ready at hand in the Cheltenham waters; our patient was generally recommended to go thither, where he seldom failed to complete his recovery in the short space of four or six weeks. Vast numbers of cases of this description have come to my knowledge.

Of late years, however, as I before remarked, a patient under similar circumstances would not think it necessary to submit his case to any preliminary treatment, but would at once proceed to Cheltenham. Your sickly, jaundiced, and deeply-damaged orientals, on their return from their baneful presidencies to England, will frequently act in this way, and when once at Cheltenham, will instantly begin their own cure by means of the water, and the water principally,—in the expected good effects of which, however, they are disappointed. How could it be otherwise? Failure indeed might have been expected; for the Cheltenham water *per se* is incapable of curing any disorders (except indeed some slight cases of indigestion,) though admirably calculated to assist in completing the cure of almost every disease and functional malady of the organs of digestion.

Viewed in this light, Cheltenham offers an immense resource to the medical practitioner; and thus recommended to invalids and convalescents, Cheltenham may be certain of a constant and vast concourse of visitors, who will there find what they require—health—and will be pleased and praise Cheltenham accordingly." 302.

We believe, however, that very few invalids from tropical or other climates, proceed to Cheltenham, without consulting some physician before they go there, and without acting under the direction of a medical man on the spot. We presume that the physicians of Cheltenham are quite capable of prescribing remedies as well as waters.

The PLUMBO-PUMPO-PHOBIA we have already disposed of in the Harrogate part of this article. The disease haunts Dr. Granville wherever he goes. The sight of six cocks in the Montpellier Rotunda nearly sets him into hysterics, and when he gets his breath, he exclaims—

"Now all this display calls largely upon the faith and credulity of the bibbers. It is not thus that matters are managed in Germany; for there the *good, honest,* and *unsophisticated people of the country* could not be persuaded to swallow *a single drop of any water which should be presented to them in so mysterious a manner, or the source of which they could not plainly see.* Here, on the contrary, I firmly believe, that were the enumeration of the taps or spouts to be carried out to three figures of numbers, there would be found people enough to drink, and feel convinced at the same time that they drank different waters." 292

Indeed! What! has Dr. Granville, in all his peregrinations, never visited the Fontaine Elysée at Aix-la-Chapelle, where the waters are delivered by two cocks to thousands and thousands annually of eager bibbers ("good, honest, and unsophisticated people") who never did, and never will see the "source" of the medicinal springs? These waters run a considerable way from their sources, through the dreaded and denunciated "pipes," which Dr. Granville kindly overlooks, although they are in Germany! And in what way could the valuable waters of Cheltenham be raised from a depth of 50 to 100 feet, but by pumps? In what way could they be transported from Lansdown Road to the Laboratoy, or to the Rotunda, but by pipes? Would Dr. Granville leave them to lie on their beds of Lias to the end of time, rather than employ a pipe or a pump to bring them within the reach of suffering mortals? And what does he dread? A nonentity! Dr. Christison has shewn that lead cannot exist in saline waters. We suspect that we shall hear no more of the "Plumbo-pumpo-phobia."

"Upon mature and deliberate reflection I hold that at the Montpellier Rotunda, the invalid who is sent to Cheltenham to drink its peculiar mineral water, will find it in perfection by using the old No. 4; or even 5, where a small quantity of iron added to the saline, is not incompatible with his complaint." 295.

It is the poor "Cambray Chalybeate" that has found least favour in the Doctor's eyes, because it is not as pregnant with carbonic acid gas as the Boklet or Bruckenau; and yet it contains twice as much of that sparkling gas as any other spa at Cheltenham.

"But to render it a paramount source of general patronage and important results, like the chalybeates in Germany, it should, like them, be sparkling with a profusion of carbonic-acid gas, instead of lying, as it does, flat and stale at its source, with its heavy mineral." 288.

Steel, however, though held in solution by a very moderate amount of carbonic acid, often proves extremely useful. Nay, where it is not in solution at all—for instance the carbonate—its medicinal efficacy is proved by the experience of mankind in all countries. The Cambray Chalybeate is not to be despised because it contains only three cubic inches of carbonic acid gas in the pint.

The Cheltenham waters may be ranged or classed under three heads—the Saline—the Sulphureous—and the Chalybeate. They all contain saline matters, and all exhibit carbonic acid gas. It must have been observed, on looking over the analyses, that muriate of soda is at the head of the saline ingredients—and next to that are the sulphates of soda, and of magnesia. Though they present several other substances, it is to the above that they owe their virtues. The purest salines are Nos. 1 and 4, at the Old Well—No. 4, at the Montpellier—and the waters of Pittville and Cambray.

These, therefore, are the waters most used in all ordinary cases of disordered stomach, liver, or bowels—in dyspepsia, nephritic complaints, dropsical affections, uterine irregularities—and in some cases of gout and rheumatism. These saline waters open all the secretions, as well as the bowels, and thus prove eminently *alterative*. Where it is judged prudent to combine with these waters a light tonic, the No. 4, A. of the Montpellier answers the purpose well.

Of the sulphureous waters we shall not say much, as they certainly appeared to us much less malodorous than those of Harrogate, or even of Leamington. The Cheltenham physicians, however, maintain that these springs, especially No. 5, at the Old Well, and No. 2, at the Montpellier, are little if at all inferior to the waters of Harrogate.

"Many persons have been inclined to doubt their efficacy, from the circumstance of their saline ingredients depriving them of the strong sulphureous taste and smell possessed by the waters of Harrogate; but this is a mistake which experience will correct, and to all those afflicted by cutaneous diseases, scrofula in its various forms, ulcers, rheumatism, gout, hæmorrhoids, worms, &c. and many female complaints, we can confidently recommend these waters as a very valuable remedy, when taken as directed under the several heads of these diseases; in many cases they will effect a complete cure, and in almost all they will afford sensible relief. They do not act particularly upon the stomach or bowels, or at least it is in a very gentle manner; but they act very sensibly on the skin, kidneys, and lungs." 22.

The above is stated by an anonymous physician, but we are privately informed that the writer was a man of talent and integrity, who had much local knowledge of the waters.

The same authority avers that the chalybeates, especially that of the Cambray Spa, are not inferior in efficacy, in those cases where they are indicated, to any chalybeates in England—"not even to those of Tunbridge."

"In female complaints especially they are of infinite service in restoring suspended or perverted function, and in generally strengthening the system. In most of the forms of scrofula they are highly valuable; and, in many instances, in convalescence from diseases which have left great debility, a course of these waters is extremely useful; and in many cases they are indicated after one or two courses of the saline or sulphureous waters; but in proportion as the chalybeate waters are beneficial in those cases to which they are adapted, so are they prejudicial if improperly and incautiously taken, and may produce the very worst effects. They never should be had recourse to but under medical advice, as it is impossible for any other than a medical man to judge in what cases and constitutions they may be useful or prejudicial. As a general remark it may be observed that they seldom or ever agree with persons of active circulation, florid complexion, and sanguine temperament; or persons subject to cough, spitting of blood, determination of blood to the head, &c.; but are well adapted to cold and phlegmatic habits, where there is languid circulation, torpor of the system, &c. whenever they produce headache, flushings of the countenance, giddiness, &c. their use should immediately be discontinued. The strongest chalybeates at Cheltenham are those of the Cambray Spa, and at the Montpellier Laboratory; and are therefore best adapted for cases in which the use of steel medicines are clearly indicated, and female complaints attended by great debility, scrofula, those cases of dyspepsia in which tonics are indicated, many nervous affections, convalescence from diseases, &c. The saline chalybeate, such as No. 1, at the Montpellier Spa, and No. 3, at the Old Wells, will be found very serviceable in those cases where it is necessary to conjoin gentle purgatives with steel medicines." 23.

The Cheltenham waters may be taken at any period of the year, when the weather is mild; but when taken as means of prevention, from the middle of March till the end of October is the best season. But, in every case, medical advice should be taken on the spot, before the course is commenced.

"In all cases where they are had recourse to, it is absolutely necessary to premise one or two doses of *medicine* of some kind, for if the stomach and bowels are loaded at the time of their commencement, they will not act, and will be sure to disagree; the choice of the medicine will depend upon the circumstances of the case, and the constitution of the patient; in general a calomel pill of three, four, or five grains at bedtime, with a black draught the following morning, or simply, pills of calomel and colocynth at bedtime will suffice.

The *saline* and *sulphureous* waters should be always taken in the morning, fasting, and in such quantities or with such adjuncts as will ensure a proper effect upon the bowels; for when this does not take place, a sense of fulness, distention and swelling will be felt, with flushings of the face, drowsiness, headache, &c. The quantity usually taken is from one to two pints. It is generally requisite to add to the first glass, sometimes to both or all, a small quantity of what is termed *solution,* (which is the water concentrated by evaporation;) this is done at the discretion of the Pumper, and frequently in cases of great torpor of the alimentary canal even this is not found sufficient, and it becomes requisite to take a pill every or every alternate night on going to bed. These waters should be taken *early* in the morning; formerly, when Cheltenham was less a place of fashionable resort than it is at present, the waters used to be drank as early as six, or at the latest seven o'clock in the morning; *now* it is no uncommon thing to see the lazy votary of fashion and the pale-faced victim of the last night's ball crawl to the well to take their *first* glass at nine or even half-past, but this is not doing justice to themselves or to the waters; the most proper hours for drinking them are undoubtedly from seven to nine in the morning. Two or three half-pint glasses, or sometimes ten or twelve ounce glasses, according to circumstances, are taken, allowing an interval of about twenty minutes between each glass; and a full hour should be allowed to intervene between the *last* and breakfast; the whole of which time may be very agreeably spent in the walk, and in listening to the excellent music which is to be heard at most of the Spas. Some persons find that they cannot walk immediately after taking the waters without a feeling of giddiness; those persons should sit down for a quarter of an hour afterwards, during which the tendency would subside."—*Anonymous.*

The chalybeates here, as at other spas, may be taken at all times of the day—beginning with a morning dose, and repeating it afterwards, once or twice in the day. Simplicity in food, early hours, abstinence from fruit and vegetables, and regular exercise, are no mean auxiliaries to the waters. The internal use of all the mineral waters at Cheltenham is beneficially attended by a warm bath once or twice a week. There is excellent accommodation, in this respect, at Mr. Thompson's Establishment adjoining the Laboratory, in Bath Street. The following quotation contains judicious advice.

"Before proceeding to speak of the *special diseases* in which the Cheltenham waters have been found beneficial, it may be advisable to say a few words on a state of the system very commonly prevailing, and which, although not amounting to actual disease, inevitably, if neglected, lays the foundation of serious organic and functional affections, and is the fruitful source of acute suffering and premature decay of the vital powers; but which, if attended to in time, is very easily overcome, and in which two or three courses of the Cheltenham waters very seldom fail to effect a cure. We allude to a state of *mal-aise,* which usually commences with, or has its foundation in, *constipation of the bowels;* the bowels act perhaps daily, or once in two days, but not sufficiently; after the evacuations have for some time been defective in quantity, the *quality* of those likewise becomes altered: they are too dark or too light in colour, or they are mixed with mucus, frequently in small white threads, resembling worms; after this has continued for some time, listlessness and languor is felt, especially in a morning; the appetite fails; head-aches are occasionally, in some cases frequently, troublesome, and the patient feels universally out of order, without being able to say that he has anything in particular the matter with him. If this state is neglected, it will lay the foundation of obstinate dyspepsia, of disease of the liver, or of the mucous membrane of the stomach and bowels, according to the constitution of the individual; but if judiciously treated in its commencement, and whilst the disorder is simply one of *function,* it will very readily yield to remedies.

In these cases the patient should, in the first instance, take at bedtime, a three, four, or five grain calomel pill, according to the age and sex of the individual, followed the next morning by a black draught of salts and senna; the morning after this a course of the waters may be commenced. In these cases we should give the preference to the *pure saline,* taking from sixteen to twenty ounces every morning, with or without *solution,* according to its effects on the bowels: two or three evacuations should be produced every day; and in order to insure the evacuation of the solid as well as the watery contents of the bowels, it is very desirable every *second* night during the *first,* and perhaps the *second* course of the water, to take a pill composed of five grains of the compound extract of colocynth, with three, four, or five of the blue pill, and where there are any reasons why the blue pill is inadmissible, the compound extract of colocynth may be taken alone, or combined with three or four grains of Castile soap. The number of courses of the water required to restore the functions to their natural state will depend upon the time the disorder has lasted; when it has been of short continuance—one course of three weeks, or two of a fortnight each, may suffice; but frequently a third course may be necessary, and even a repetition of them the succeeding autumn or spring may be advisable; and in some persons the tendency to functional disorder of the stomach and bowels is so inveterate, that an annual course of the waters, nay, in some cases one or two courses each spring and autumn, is absolutely necessary to keep the system in any thing like order. Nor let the invalid in whose case this may be necessary complain, if he possesses the means of visiting this favoured spot; rather should he rejoice that nature has provided such a pleasant and health-restoring beverage, which not only preserves him in a state of comfort, and allows him the enjoyment of the blessings of this life, but also does away with the necessity for drenching his stomach with drastic purgatives, tonic bitters, carminatives, and the endless list of medicines to which a dyspeptic invalid has recourse for a temporary alleviation of his sufferings, and in lieu of them imposes upon him the pleasure of an annual visit or two to one of the most delightful watering places in the world, where every thing is combined that can heal the body, soothe the mind, delight the eye, and amend the heart." 34.

The aperient qualities of the Cheltenham waters—especially the pure saline—render them admissible in cases of plethora, or even local congestion, where the generality of other spas containing exciting ingredients, would be dangerous.

"There is another state of the system, not amounting to actual disease, in which a spring or autumnal course, annually, of the Cheltenham waters, is strikingly useful in preserving the balance of health, and warding off serious disorders; we mean that state of *plethora* to which many persons of stout make and full habit are liable. This affection is of *two* kinds: viz. *absolute* plethora, or general fulness of blood, which occurs commonly in persons of robust habits, florid complexion, full pulse, good appetite, and rather constipated habit of bowels. These cases are not only completely relieved by one or two full courses of the waters, but as there is in these persons a tendency to make blood too rapidly, and in too large a quantity, local congestions, or determinations of blood are prevented; and the absolute quantity of the mass of blood is diminished, by the saline qualities of the water acting copiously upon the exhalants of the bowels and carrying off the watery parts of the blood. When this habit of body prevails, an annual visit to Cheltenham is of essential importance, which, when joined to a moderate and rather spare diet, with regular exercise, will suffice to prevent the necessity of those frequent abstractions of blood, to which such invalids are but too apt to have recourse, and which however necessary they sometimes may be, have an inevitable tendency to re-produce the necessity for their repetition. There is another state of *plethora* which has been termed *relative*, implying not that the *quantity* of the blood is absolutely too great, but that it is so relatively to the powers of the constitution for appropriating or disposing of it. In this case the deviation from health is very gradual, and at first excites but little attention; there is languor and debility, a chilly state of the surface of the body, cold feet, and very languid circulation; the internal and large blood-vessels having thus an unusual load thrown upon them, local congestion takes place; producing, according to the part affected, head-ache, difficulty of breathing, indigestion, constipation, pains in the stomach or bowels, and alternation of flushed and pale countenance, sometimes ulcers of the legs, &c. Indeed, if this state continues long some *local ailment* is sure to arise. Persons not acquainted with the nature of this complaint, are apt to consider it as one of *pure debility*, consequently they take tonic medicines, full diet of beef-steaks and porter, port wine, &c., thus adding fuel to the disorder; whereas it must be treated, sometimes even by general or local bleeding, but always by a course of purgatives, for if in this state there is not costiveness of the bowels, there is *invariably* a very foul state of the secretions; those from the bowels are dark coloured and offensive, and the urine is high-coloured and loaded. In these cases the Cheltenham saline water, aided by the colocynth and blue-pills, are sure to effect a cure—but they require great perseverance, two courses spring and autumn, for several successive seasons being frequently necessary before the system can be brought to its natural state. We are acquainted with several instances of both these forms of plethora, where the subjects of them were in the habit of visiting Cheltenham at first twice, and now continue it regularly *once* a-year, and by this means keep themselves in perfect health; when previously to being made acquainted with the virtues of these waters in such cases, they had been in the habit of losing large quantities of blood every year, besides taking quantities of drugs of various kinds, but the necessity for which is now by the regular use of these waters done away with." 36.

It is for the Proteian forms of indigestion and biliary derangements, however, that the waters of Cheltenham are chiefly had recourse to. Cheltenham, in fact, forms a kind of valetudinarium for the tropical invalids, of both hemispheres, as well as for a numerous class of invalids who have never left the English shores, but whose digestive organs become impaired by sedentary habits, anxiety of mind, and the wear and tear of professional, commercial, and political pursuits. It is here, too, that we see hypochondriasis on a tolerably large scale. Speaking of the hepatic complaints which accumulate here from hot climates, the physician already quoted observes :—

"In these cases, especially, when they are the consequence of residence in a warm climate, a steady use of the Cheltenham waters for a considerable time, (at least two or three courses of three weeks each) and aided by the occasional remedies, will seldom fail to overcome the disorder. As usual it will be requisite to commence by one or two doses of purgative medicine. During the first course it will be desirable to take one of the colocynth and blue pills every night, and sixteen or twenty ounces of the pure saline water every morning; taking care, by the addition of solution if necessary, to ensure three or four evacuations from the bowels daily: if there is progressive amendment, the pill may be taken every *second* night only during the second course, and the water may with great propriety and advantage be changed for the No. 4 A, of the Montpellier Spa. During the third course the pills may be omitted, and the last-mentioned water taken in such quantity as to produce at least two evacuations daily."—*Anonymous.*

But our limits are already transgressed, and we cannot accompany Dr. Granville to Bath and several other places, which we have often personally explored. If we have quoted but little from Dr. Granville, he must recollect that he has emphatically stated his object to be almost entirely for popular instruction. To the general reader, especially to those who are about to visit the English spas and watering-places, the volumes of our lively and amusing author will be most welcome and instructive, as they are, in fact, excellent hand-books for the mineral waters. But to the profession they will be much less valuable, in consequence of the details and descriptions which render them attractive to the non-professional traveller.

